# Long-term female bias in sex ratios across life stages of Harpy Eagle, a large raptor exhibiting reverse sexual size dimorphism

**DOI:** 10.1098/rsos.231443

**Published:** 2023-11-08

**Authors:** Aureo Banhos, Tânia Margarete Sanaiotti, Renan Coser, Waleska Gravena, Francisca Helena Aguiar-Silva, Mylena Kaizer, Tomas Hrbek, Izeni Pires Farias

**Affiliations:** ^1^ Departamento de Biologia, Centro de Ciências Exatas, Naturais e da Saúde, Universidade Federal do Espírito Santo - UFES, Alto Universitário, s/n°, Guararema, 29500-000 Alegre, Espírito Santo, Brazil; ^2^ Programa de Pós-Graduação em Ciências Biológicas (Biologia Animal) - PPGBAN, Universidade Federal do Espírito Santo - UFES, Avenida Fernando Ferrari, 514, Prédio Barbara Weinberg, 29075-910 Vitória, Espírito Santo, Brazil; ^3^ Projeto Harpia (Harpy Eagle Project - Brazil), Instituto Nacional de Pesquisas da Amazônia – INPA, Av. André Araújo, 2936, Aleixo, 69067-375 Manaus, Amazonas, Brazil; ^4^ Projeto Harpia – Mata Atlântica (Harpy Eagle Project - Atlantic Forest), Universidade Federal do Espírito Santo - UFES, Alto Universitário, Guararema, 29500-000 Alegre, Espírito Santo, Brazil; ^5^ Laboratório de Evolução e Genética Animal - LEGAL, Universidade Federal do Amazonas - UFAM, Av. General Rodrigo Octavio Jordão Ramos, 6200 - Coroado I, 69080-900 Manaus, Amazonas, Brazil; ^6^ Programa de Pós-Graduação em Genética, Conservação e Biologia Evolutiva, Instituto Nacional de Pesquisas da Amazônia - INPA, Av. André Araújo, 2936, Aleixo, 69067-375 Manaus, Amazonas, Brazil; ^7^ Coordenação de Biodiversidade, Instituto Nacional de Pesquisas da Amazônia - INPA, Av. André Araújo, 2936, Aleixo, 69067-375 Manaus, Amazonas, Brazil; ^8^ Instituto de Saúde e Biotecnologia, Universidade Federal do Amazonas - UFAM, Estrada Coari Mamiá, 305, Espírito Santo, 69460-000 Coari, Amazonas, Brazil; ^9^ Programa de Pós-Graduação em Zoologia - PPGZOO, Universidade Federal do Amazonas - UFAM, Av. General Rodrigo Octavio Jordão Ramos, 6200, Coroado I, 69080-900 Manaus, Amazonas, Brazil; ^10^ Departamento de Genética, Instituto de Ciências Biológicas, Universidade Federal do Amazonas - UFAM, Av. General Rodrigo Octavio Jordão Ramos, 6200, Coroado I, 69080-900 Manaus, Amazonas, Brazil; ^11^ Department of Biology, Trinity University, San Antonio, TX 78212, USA

**Keywords:** evolution, Fisher's principle, life history, molecular sexing, sex allocation, sex roles

## Abstract

The primary (PSR), secondary (SSR) and adult (ASR) sex ratios of sexually reproducing organisms influence their life histories. Species exhibiting reversed sexual size dimorphism (RSD) may imply a higher cost of female production or lower female survival, thus generating biases in PSR, SSR and/or ASR towards males. The Harpy Eagle is the world's largest eagle exhibiting RSD. This species is found in the Neotropical region and is currently threatened with extinction. We used molecular markers to determine the sex of 309 Harpy Eagles spanning different life stages—eaglets, subadults and adults—from 1904 to 2021 within the Amazon Rainforest and Atlantic Forest. Sex ratios for all life stages revealed a female-biased deviation across all periods and regions. Our results suggest that the population bias towards females is an evolutionary ecological pattern of this species, and SSR and ASR likely emerged from the PSR. This natural bias towards females may be compensated by an earlier sexual maturation age of males, implying a longer reproductive lifespan and a higher proportion of sexually active males. A better understanding of the Harpy Eagle's life history can contribute to understanding sex-role evolution and enable more appropriate conservation strategies for the species.

## 1. Introduction

The ratios between males and females at fertilization and conception (primary sex ratio—PSR), birth (secondary sex ratio—SSR) and during adulthood (adult sex ratio—ASR) influence life histories of sexually reproducing organisms [1,2]. Sex allocation theory assumes that natural selection favours parents who modify their investment in male and female offspring to maximize their fitness [1,3]. However, as long as males and females are equally costly to produce, unbiased PSRs and SSRs are expected in the population, because frequency-dependent selection favours an equal number of offspring for both sexes [1]. On the other hand, there are specific predictions regarding the balance between PSR and SSR for a variety of scenarios, with differential costs allocated toward male and female production [3–7]. Resource availability, maternal condition and quality, male attractiveness or quality, social behaviour, sibling competition and sexual conflict are some of the factors that predict differences in offspring sex allocation ratios [5,8–10], but there is no obvious mechanism by which parents can manipulate PSR and SSR [11–13]. However, in species that exhibit parental care, the manipulation of the offspring sex ratio (SR) is possible through differential investment in males and females during the parental care period until juvenile independence [9].

By contrast, for ASR, there are no predictions, such as those for PSR and SSR, because frequency-dependent selection does not operate on ASR [14]. However, ASR regulates changes in intraspecific traits, through sexual selection of the reproductive system and sexual dimorphism of species, that influence maturation [15,20]. ASR emerges as a result of sex-biased PSR and SSR, specific processes affecting SRs at other stages of the life cycle, such as maturation times, differential mortality of juvenile and adult individuals and sex-biased dispersal and migration patterns [14,16–20]. In general, birds and mammals present a balanced PSR, but they become biased during their adult life owing to differential sex-specific mortality, which is higher for females among birds and higher for males among mammals [21]. This sex-specific mortality is due to the genetics of sex determination because mortality is higher in the heterogametic sex [19,21], as predicted by Haldane's rule [22,23]. Some bird species threatened with extinction have a positive male bias [24], which has implications for their conservation [25].

Bird and mammal species with sexual size dimorphism (SSD) illustrate the SR theory [26,27]. In this case, if available resources are limited, offspring of the larger sex may receive relatively fewer resources, thus compromising their development and survival [28–31]. Parents may respond by adjusting the production of offspring of the smaller size, which generates a bias in PSR and SSR [31–34]. In addition, bias initiated in the SSR can be increased or decreased by sex-biased juvenile mortality, leading to biased ASR [18]. This biased mortality often affects the sex of the larger size, as it is more sensitive to food scarcity and variation in other ecological factors [18,35,36]. Biased ASR can generate strong competition for partners among individuals of the rarer sex, thereby generating SSD [20,37,38].

Species that exhibit reversed sexual size dimorphism (RSD), where females are larger than males, may have different consequences than SSD on sexual roles and present an interesting case. However, few studies have addressed this issue. RSD suggests a higher cost of female production or lower female survival, which generates a bias in the PSR, SSR and ASR towards males [33,39–42]. However, some studies with raptors have found a bias towards females [43,44] and/or no bias [41,45]. In raptors, RSD is adaptive and likely evolved for nest and offspring protection, with larger females being able to better defend and care for offspring [46].

**Figure 1.**
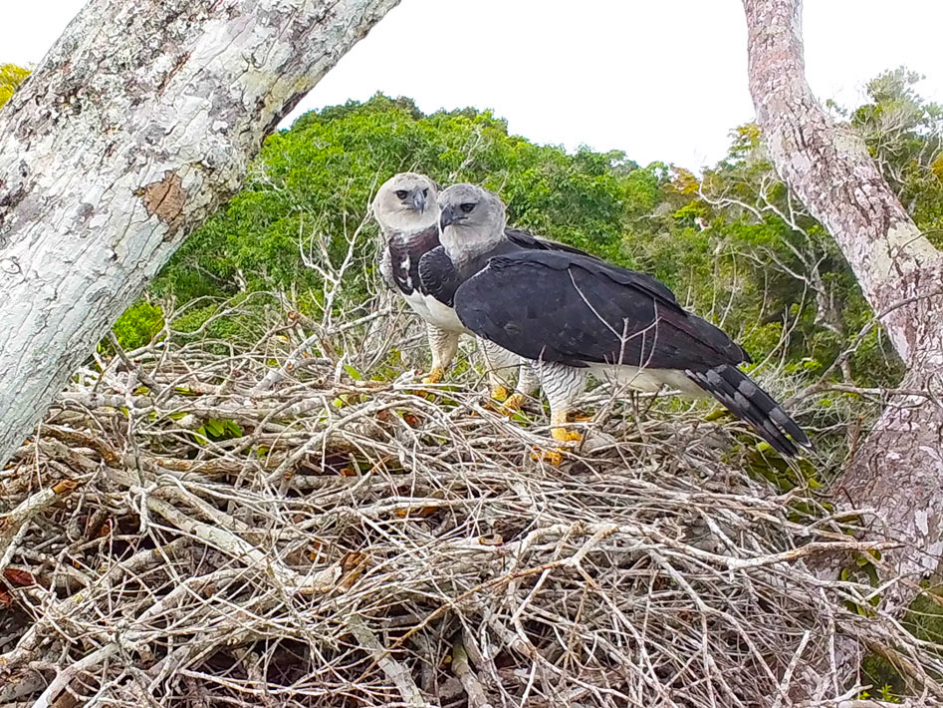
A breeding pair of Harpy Eagle in a nest in the Veracel Station Private Natural Heritage Reserve, Bahia, Brazil. Photo by Harpy Eagle Project—Atlantic Forest.

The Harpy Eagle, Harpia harpyja (Linnaeus, 1758) (figure 1) is one of the world's largest eagles. It exhibits RSD in body size. Adult and subadult males weigh between 4.1 and 4.8 kg, while females weigh between 5.8 and 7.6 kg [47–49], with females being 21–85% heavier than males. Furthermore, adult males have wings measuring 543–580 mm, while females have wings measuring 583–610 mm [50]. Harpy Eagles are found in Neotropical forests from southern Mexico to northeastern Argentina and eastern Brazil [51–53]. This species depends on forests for survival because its diet mainly consists of arboreal prey [54–59]. Pairs nest in emergent canopy trees and usually return to the same tree for reproduction [48,60–62]. They produce one eaglet every 2.5–3 years, which is provided with long parental care [60,63,64]. Eaglets begin flying at around four to six months of age and remain in their parents' territory for another 2 years, receiving parental care before dispersing [60,63,65,66]. They do not reach full adult plumage before 4–5 years of age [48], which is close to their age of reproductive maturity [67]. Individuals have a long fertile lifespan of over 35 years [67] and a long generation time of 20 years [53]. Adults have large home ranges [49,68], with area required by each pair of 10–79 km^2^ [54,63,69,70]. The decline in their population owing to intense habitat loss and the removal of individuals from the wild by hunting and persecution has threatened the Harpy Eagle with extinction and placed it in the Vulnerable category throughout its distribution area [53].

Despite the Harpy Eagle presenting RSD, it does not present sexual dimorphism in plumage (figure 1), which challenges studies on sexual roles in this species. Visually, males and females are not easily distinguishable from a distance or when they are eaglets and subadults. Individuals are difficult to detect in dense forests, and even when they are found, they are extremely difficult to observe, capture and mark. One way to minimize these limitations is to employ indirect research techniques, such as molecular genetic analyses of shed feathers from wild individuals, and of samples of specimens deposited in museums and captive-held individuals [71–73]. In this study, we used molecular markers to determine the sex of Harpy Eagle individuals from a century's worth of samples collected from individuals widely distributed in the Amazon Rainforest and Atlantic Forest regions to analyse the SRs of eaglets, subadults and adults.

## 2. Methods

### 2.1. Sampling

**Figure 2.**
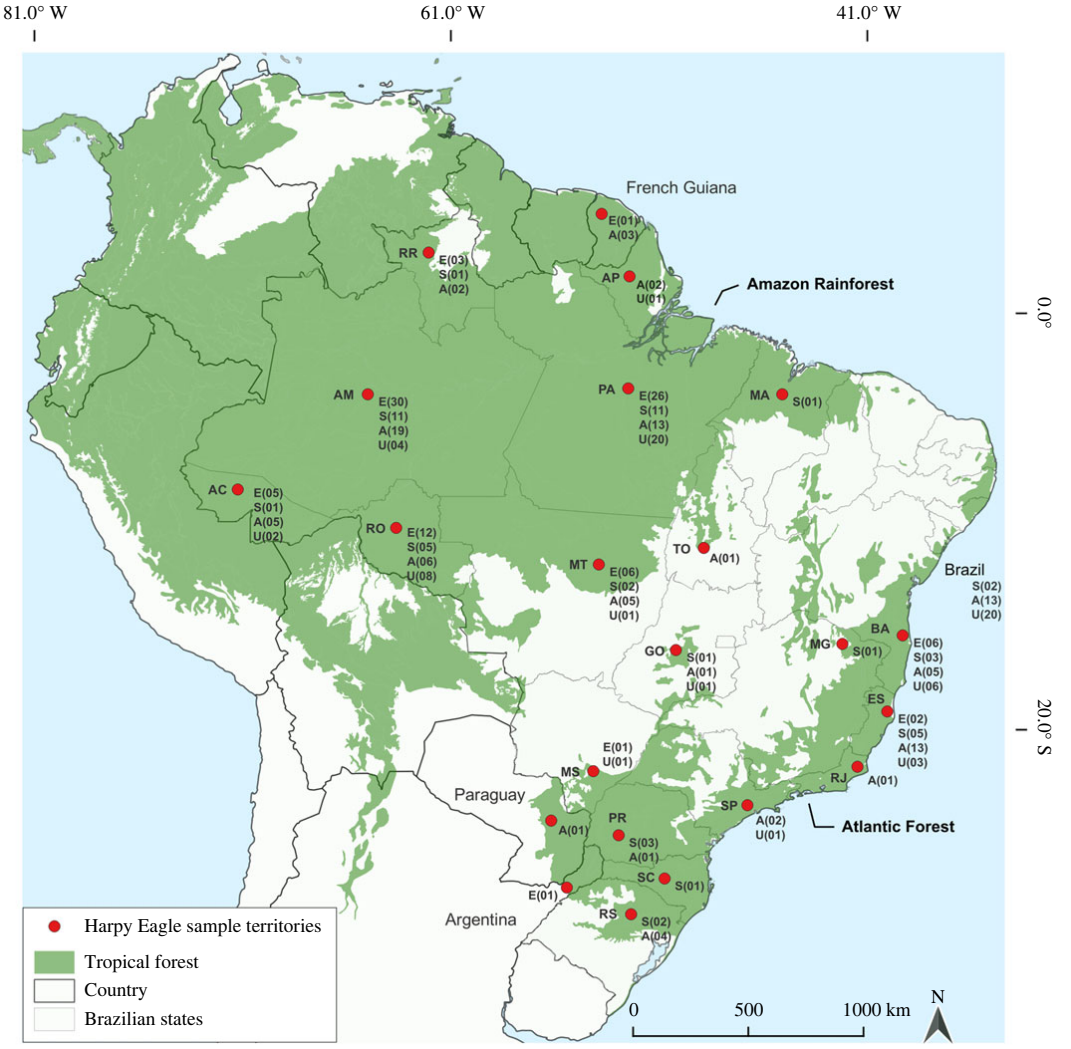
Area where Harpy Eagles were sampled in the Amazon Rainforest and Atlantic Forest in the states of Brazil (AC, Acre; AP, Amapá; AM, Amazonas; BA, Bahia; ES, Espírito Santo; GO, Goiás; MA, Maranhão; MT, Mato Grosso; MS, Mato Grosso do Sul; MG, Minas Gerais; PA, Pará; PR, Paraná; RS, Rio Grande do Sul; RJ, Rio de Janeiro; RO, Rondônia; RR, Roraima; SC, Santa Catarina; SP, São Paulo and TO, Tocantins), and in the Paraguay, Argentina and French Guiana. It shows the number (*n*) of eaglets—E, subadults—S, adults—A and individuals in an unidentified developmental stage—U per political territory.

We analysed samples from 309 Harpy Eagle individuals (electronic supplementary material, table S1) collected between 1904 and 2021 from the forests of Brazil, as well as Argentina, French Guiana and Paraguay, covering regions in the domains of the Amazon Rainforest and Atlantic Forest (figure 2). A total of 243 feather, 43 blood and 23 tissue samples (muscle, liver, skin and bone) were used as DNA sources. Samples were obtained from individual eaglets from known nests, free-living individuals (living in the wild), rescued individuals kept at breeding grounds, and dead individuals deposited in museum collections (removed from the wild). Of these, 211 were from the Amazon Rainforest, 63 were from the Atlantic Forest and 35 were from Brazil, but of unknown origin (electronic supplementary material, table S1 [74]). Nesting adults were not included in the sampling because the SR of the pairs is 1 : 1, and there may be a greater presence of adult female feathers in the nests because the adult female spends more time in the nest to take care of the eaglets.

### 2.2. Classification of an individual's life stage

**Figure 3.**
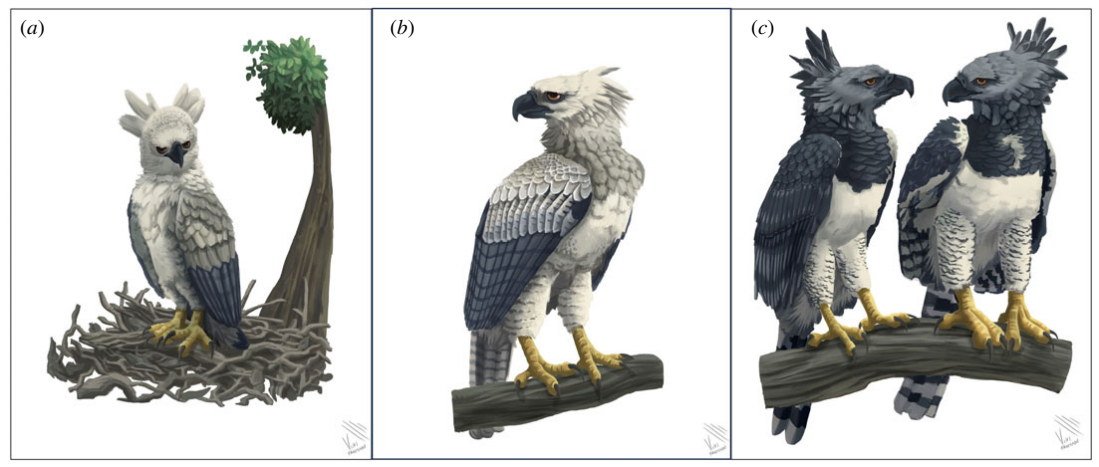
Life stage of the Harpy Eagle: (*a*) eaglet; (*b*) subadult and (*c*) adults: on the left, the male, on the right, the female.

Eaglets (figure 3*a*): We examined samples from 93 nestlings and nest-dependent eaglets up to 2 years of age, collected from individuals associated with nests (electronic supplementary material, table S1 [74]). All had a known birth year, between 1968 and 2021. For individuals removed from the wild with information associated with a nest, the year of removal was considered to be the year of birth. Some eaglets were born in the same nest in different years. Thus, the eaglets were divided into those born in the same nest with more than one reproductive cycle sampled (*n* = 45 in 19 nests: 14 nests with two eaglets, three nests with three eaglets and two nests with four eaglets born in different reproductive cycles) and those born in different nests with one reproductive cycle sampled (*n* = 48). There were 83 eaglets were from the Amazon Rainforest and 10 from the Atlantic Forest. Overall, there were 71 feather samples, 16 blood samples and six tissue samples.

Subadults (figure 3*b*): We examined 50 individuals with non-adult plumage ranging from approximately 2–4 years of age (electronic supplementary material, table S1 [74]): 33 subadults from the Amazon Rainforest, 15 from the Atlantic Forest and two from unidentified regions. They were either removed from the wild alive or dead, or deposited in museums (removed, *n* = 47). We also considered fallen feathers with non-adult coloration to be from wild individuals (*n* = 3). To distinguish developmental phase by plumage, we used the information from second to fourth subadult plumages of the Harpy Eagle (17–50 months old) provided by Fowler & Cope [48] and reference data gathered by the Harpy Eagle Project. We determined the collection year off all but one individual. In total, there were 36 feather samples, 11 blood samples and three tissue samples.

Adults (figure 3*c*): We examined 98 individuals with full adult plumage older than 5 years (electronic supplementary material, table S1 [74]): 58 adults from the Amazon Rainforest, 28 from the Atlantic Forest and 12 from unidentified regions. They were either removed from the wild alive or dead, or deposited in museums (removed, *n* = 83). We also considered the fallen feathers found in the wild with an adult pattern as originating from adults (*n* = 15). We could only determine the collection year for 85 individuals. In total, there were 80 feather samples, 12 blood samples and six tissue samples.

Unidentified life stage: We examined 68 individuals with unidentified developmental stages (electronic supplementary material, table S1 [74]): 37 from the Amazon Rainforest, 10 from the Atlantic Forest and 21 from unidentified regions. They were either removed from the wild alive or dead, or deposited in museums (*n* = 63). Some samples were fallen feathers collected in the wild, whose stage of development could not be determined (*n* = 5). We were only able to determine the collection year for 41 individuals. In total, there were 56 feather samples, four blood samples and eight tissue samples.

### 2.3. DNA extraction

We extracted DNA using a Qiagen DNeasy Blood & Tissue Extraction Kit. To degrade keratin in the feathers, we added 30 µl of dithiothreitol (100 mg ml^−1^) in the first step of the protocol, based on the method described by Horváth *et al*. [75]. For some of these samples, we adopted the modifications of Peters *et al*. [76] in the protocol, which consisted mainly of increasing the incubation time to 48 h.

### 2.4. Molecular sexing

The samples were sexed with the primers NP, CHD1Wr and CHD1Zr following Banhos *et al*. [71]. Molecular methods to determine sex are based on the amplification of intron of the paralogous copies of the Chromodomain Helicase DNA binding protein (CHD1) gene present in sex chromosomes [77]. In birds, the CHD1 gene, present in both the W and Z sex chromosomes, was amplified using mismatched primers. In other words, while one of the primers (primer NP) amplified both paralogous copies of the CHD1 gene on the W and Z chromosomes, the other pair was designed to anneal specifically to the W (primer CHD1Wr) or Z (primer CHD1Zr) chromosome. The NP/CHD1Wr primer pair amplifies a larger product, whereas the NP/CHD1Zr primer pair amplifies a smaller product; the homogametic sex (male) is identified by the presence of a single approximately 300 bp fragment, while the heterogametic sex (female) is identified by the presence of two fragments, one approximately 300 bp and the other approximately 250 bp. By designing the CHD1Z fragment to be smaller than the CHD1W, we eliminated the possibility of identifying females as males in highly degraded samples (non-amplification of the smaller CHD1Z will not occur if the larger CHD1W amplifies, and if only the smaller CHD1Z fragment amplifies, that specimen can be confidently attributed to male) [71].

We performed the polymerase chain reactions (PCRs) for the markers at a total volume of 25 µl containing 1 µl of DNA (5–30 ng), 2.5 µl of NP primer (2 µM), 1.3 µl of CHD1Zr primer (2 µM), 1.3 µl of CHD1Wr primer (2 µM), 2.5 µl of 10× buffer (Tris-KCl 200 mM, pH 8.5), 2.5 µl MgCl_2_ (25 mM), 2.0 µl dNTP (10 mM), 0.2 µl Taq DNA polymerase (5 U ml^−1^) and 11.8 µl of deionized water. The thermocycling profile consisted of an initial denaturation at 93°C for 1 min, followed by 35 denaturation cycles at 93°C for 10 s, primer pairing at 50°C for 35 s and DNA strand extension at 68°C for 30 s. The reaction was completed by final extension for 7 min at 68°C. The PCR products were separated on a 3% (w/v) agarose gel containing 10 µl of PCR product. To confirm the results, PCRs were performed two to five times per sample, genotyping 226 samples using this method. Degraded and low-quality DNA (a common situation for feather samples collected non-invasively) was amplified with greater difficulty. Therefore, it was not possible to identify sex or determine it with confidence in some of the eight feather samples; these samples were not included in this study.

To better visualize the fragments with small concentrations in the PCR product, we added a M13 tail to the NP primer and used the M13-FAM-6 primer [78] along with other PCR reagents. The PCR conditions and thermocycling were adapted according to Schuelke [78] for a total volume of 10 µl of the PCR product. The PCR products were diluted, and 8 µl of Hi-Di formamide (Applied Biosystems, Inc.) and 1 µl of 6-Carboxy-X-rhodamine (ROX), a standard size marker used for comparison with the PCR fragments, were added to each PCR product [79]. Subsequently, we ran the products on an ABI 3130XL sequencer following the manufacturer's protocol. We visualized the genotypes using GeneMapper software (v.4.0; Applied Biosystems Inc.) to infer the allele sizes at each locus. Genotyping was repeated in cases in which the genotypes were not clearly observed in the first attempt. A total of 83 samples were genotyped using this method.

### 2.5. Sex ratio estimates

We considered the following groups in our analyses of the SR:

1. Life stage: 1.1. eaglets; 1.2. subadults; 1.3. adults; and 1.4. unidentified.
2. Eaglets: 2.1. nests with one reproductive cycle sampled; and 2.2. nests with multiple reproductive cycles sampled.
3. Life stages by region: 3.1. eaglets from the Amazon Rainforest; 3.2. subadults from the Amazon Rainforest; 3.3. adults from the Amazon Rainforest; 3.4. eaglets from the Atlantic Forest; 3.5. subadults from the Atlantic Forest; and 3.6. adults from the Atlantic Forest.
4. Life stage by temporal period: 4.1. eaglets (1968–2006); 4.2. eaglets (2007–2021); 4.3. subadults (1923–1964); 4.4. subadults (1983–2006); 4.5. subadults (2007–2021); 4.6. adults (1904–1961); 4.7. adults (1970–2006); 4.8. adults (2007–2021).

Additionally, we checked the SR in the samples of eaglets, subadults and adults removed from the wild that were deposited in museums and zoos, as well as those of free-living individuals. We also examined the Harpy Eagle sex ratio based on the type of samples examined: feather and tissue.

SR was expressed as the proportion of males (SR = males/(males + females)) and deviations were expected to conform to a binomial distribution [80]. We tested whether the SR deviates significantly from parity by using generalized linear models (GLMs) with a standard error of binomial distribution and the logit-link function in the lme4 package in R [81] and a modification of the script by Eberhart-Phillips *et al*. [82]. We assumed the confidence interval of 0.95 to verify whether the proportion of males was within the expected range. To compare the SRs between the groups, we adopted the log-odds scale, with a confidence interval of 0.95, and Tukey's test in the emmeans package in R. A value of *p* ≥ 0.05 was considered not to be significant.

## 3. Results

**Figure 4.**
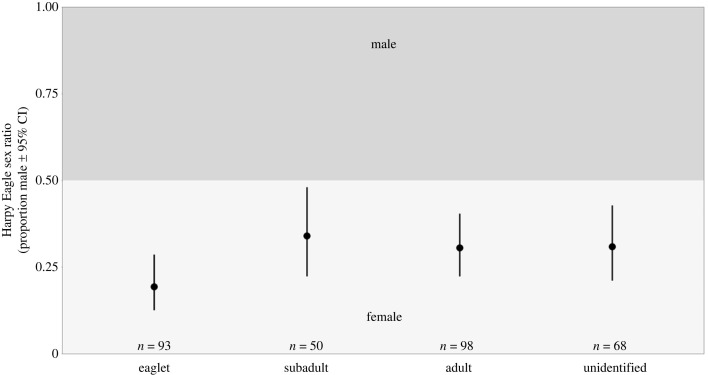
Harpy Eagle sex ratio by life stage: eaglets, subadults, adults and individuals in an unidentified developmental stage.

**Figure 5.**
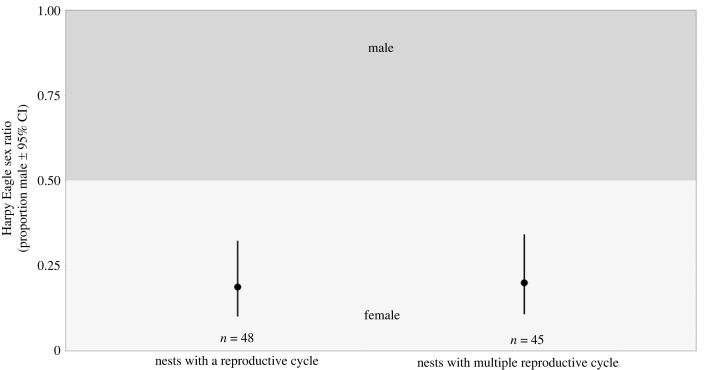
Eaglet Harpy Eagle sex ratio: nests with one reproductive cycle and nests with multiple reproductive cycles sampled.

**Figure 6.**
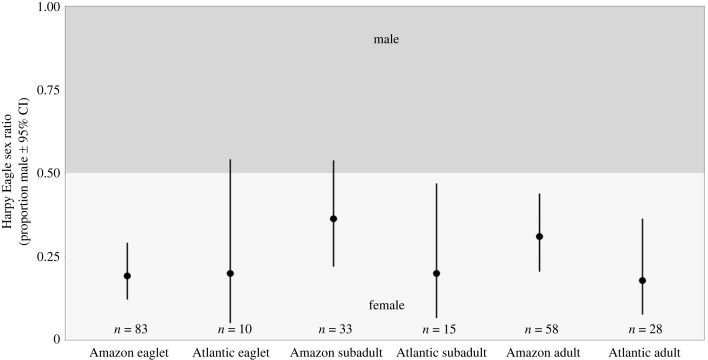
Harpy Eagle sex ratio by region: eaglets from the Amazon and Atlantic forests, subadults from the Amazon and Atlantic forests, and adults from the Amazon and Atlantic forests.

**Figure 7.**
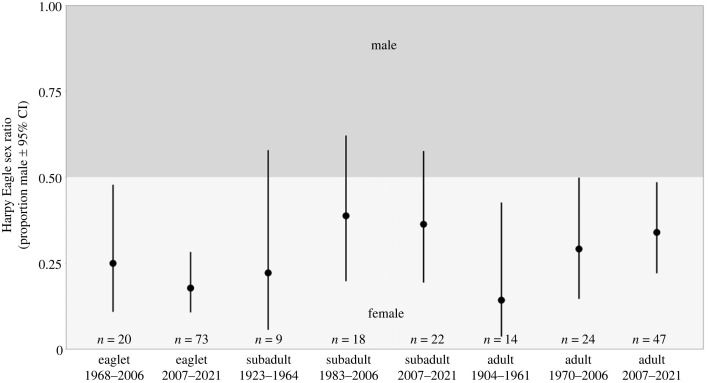
Harpy Eagle sex ratio by temporal period: eaglets (1968–2006), eaglets (2007–2021), subadults (1923–1964), subadults (1983–2006), subadults (2007–2021), adults (1904–1961), adults (1970–2006) and adults (2007–2021).

The SR of eaglets in the nest exhibited a female bias, a trend that persisted among the subadults upon independence and into adulthood (SR = 0.19, 0.34 and 0.31, respectively; figure 4). A greater proportion of females was also observed among samples with unidentified life stages (SR = 0.31; figure 4). Notably, SRs of the eaglets from nests with one reproductive cycle sampled and those with multiple reproductive cycles sampled both significantly skewed towards females (SR = 0.19 and 0.2, respectively; figure 5). Furthermore, SRs of eaglets and adults from the Amazon Rainforest (SR = 0.19 and 0.31, respectively), as well as subadults and adults from the Atlantic Forest (SR = 0.2 and 0.18, respectively) demonstrated significant female bias, with eaglets from the Atlantic Forest (SR = 0.2) and subadults from the Amazon Rainforest (SR = 0.36) also skewed towards females (figure 6). Examining temporal periods, SRs of all eaglets (SR = 0.25 and 0.18, of 1968–2006 and 2007–2021 groups, respectively) and adults (SR = 0.14, 0.29 and 0.34, of 1904–1961, 1970–2006 and 2007–2021 groups, respectively) leaned significantly towards females, while all subadult (SR = 0.22, 0.39 and 0.36, of 1923–1964, 1983–2006 and 2007–2021 groups, respectively) displayed a female bias (figure 7). Despite SRs fluctuating across the groups, no significant differences were identified in SR between the groups in any of the comparisons (electronic supplementary material, table S2 [74]).

A greater proportion of the free-living Harpy Eagle feathers found in the wild belonged to females (SR = 0.2; electronic supplementary material, figure S1 [74]). Furthermore, Harpy Eagle females were more frequently deposited in museums and zoos than males (SR = 0.32; electronic supplementary material, figure S1 [74]). The higher proportion of females was also consistent for both the tissue and feather samples of Harpy Eagle (SR = 0.23 and 0.29, respectively; electronic supplementary material, figure S2 [74]). These results demonstrate that female bias in the Harpy Eagle's sex ratio was not influenced by sample source or type.

## 4. Discussion

We obtained consistent results on SSR and ASR of a large eagle species exhibiting RSD. DNA samples were obtained from wild Harpy Eagles over the course of more than 100 years across a wide geographical distribution. Our sampling was spatially and temporally comprehensive for a species with a low population density, although it presented limitations for comparison. It was not possible to compare the number of eaglets with subadults and adults in the historical museum samples. However, many individuals considered to be subadults in the museum samples may have been eaglets that were approximately 2 years old. Because there was no information that they were collected from the nest, they were classified as subadults. However, we obtained robust sampling of eaglets in nests over the last 20 years. Our results suggest that SSR bias is an evolutionary ecological pattern in Harpy Eagles. Nests with more than one reproductive event did not balance the SR, and maintained the same pattern of bias in female production found in nests with only one reproductive event. We did not investigate PSR, because it requires invasive access and handling of eggs, but SSR likely is a consequence of it. Although the female RSD observed in birds of prey may be more costly to produce, Harpy Eagles invest more in female production. Understanding the function and cause of this pattern is challenging, but is likely closely tied to the specifics of the species' life history.

### 4.1. Harpy Eagle SSR

Harpy Eagles lay up to two eggs, with a long interval of 4–9 days between the first and second eggs being laid [60,83–85]. Harpy Eagles eggs can vary in size [83]. However, it is not known whether there is an association between egg size and offspring sex or the time interval between the first and second eggs being laid and the offspring sex. It takes approximately 56 days for the mother to incubate the eggs; in rare instances, the male assumes this role [60,84]. In general, only one eaglet hatches when two eggs are laid [60,84], but it is unknown whether a relationship exists between laying order and hatching order, and between laying order or hatching order and offspring sex. Species that exhibit sexual dimorphism in eggs can manipulate sex at birth [86]. We do not know if there is sexual dimorphism in Harpy Eagle eggs, but it seems unlikely to be so. Additionally, the female Harpy Eagle may eat their own eggs and even their offspring [84]. However, the SRs at birth found in studies of captive Harpy Eagles did not deviate from 1 : 1 [67,87]. Watson *et al*. [67] recorded the births of 49 Harpy Eagles in zoos in Central America: 25 males and 24 females. Oliveira *et al*. [87] also recorded the births of 53 Harpy Eagles in zoos: 29 males and 24 females. But the number of males (*n* = 35) that entered zoos from the wild was lower than the number of females (*n* = 51) [87]. Zoos maximize the production of Harpy Eagle eaglets by removing and artificially incubating the eggs and caring for them. Moreover, egg removal induces females to lay a second or even a third egg [67]. Whether or not a relationship exists between sex and egg size or laying order is a question that can be answered experimentally in zoos that breed Harpy Eagles rather than in the wild.

Weights of developing Harpy Eagle eaglets associated with sex begin to appear after the second week and became more pronounced around the fourth month of their development in captivity [88]. This is the same age at which wild eaglets have been reported to begin flying [60,63,65,66]. Differences in offspring care requirements may arise during this period of differentiation, such as the greater costs involved in rearing the larger sex [31]. During the two months of egg-laying and incubation, the Harpy Eagle male brings prey to the female. After the eaglet hatches, the mother spends another two months watching the nest, protecting the eaglet from the elements (rain and sun), and feeding it with prey exclusively hunted by the father [60]. After females resume hunting, they provide more parental care [60] and can capture larger prey than males [58,62]. There may be little or no sexual conflict between parents in producing more females until the first months of life. However, it is important to investigate whether there is a difference in the investment of both parents in parental care of male and female offspring until their independence.

Female Harpy Eagle eaglets may take longer to develop and spend more time receiving care than male eaglets [67,89]. This could cause differential survival between the sexes in the wild, with higher mortality for larger-sized sexes since they require more parental care [90]. The proportion of male Harpy Eagle eaglets (19.35%) was smaller than that in the following life stages (34% and 30.61% for subadults and adults, respectively), but the difference was not significant. We could think that eaglet females have higher mortality rates, which would increase the proportion of males at the age of independence. Nonetheless, we know that eight eaglets died in the nest from natural causes, of which five were females (62.5%) and three males (37.5%). Although males appear to die more than their proportion of the eaglet population, the proportion of males increases in the population after leaving the nest. This may be the effect of males leaving the nests earlier and maturing earlier than females, entering a greater proportion the population of subadults and adults than eaglets (see below).

### 4.2. Harpy Eagle ASR

We must also note a bias in the adult SR [2], as this may play an important role in the life history of the species [14,16,25]. Female-biased ASR in the Harpy Eagles emerged directly from the SSR. This differs from the situation observed in deviations for birds, which presents a bias towards males due to the higher mortality of females in most cases [21]. With a more biased ASR, sex in excess numbers may experience more evolutionary pressure due to intrasexual competition for breeding resources, such as partners and nesting sites, generating consequences in sex roles in birds and mammals [17,18,25]. For example, in shorebird populations with biased ASRs due to mortality, parental cooperation in the care of offspring may break down and the brood is then cared for by a single parent [82]. In the case of excess females, parental care is expected to be provided primarily by females in birds and mammals [16,18,25,91]. However, since the RSD trait in raptors likely evolved because of nest protection, with larger females promoting greater care for offspring [46], an excess of females in the adult raptor population would not be the primary cause of greater parental care by females in eagles. Furthermore, female-biased ASR in Harpy Eagles could reinforce their investment in parental care. Nevertheless, RSD may be a more important predictor of the life history of raptors [46] than ASR.

The causal role in the evolution of sex roles does not lie in the ASR per se [25]; it lies in the sex ratio at maturation (MSR) or in the higher uniform mortality of one of the adult sexes in parental care and in competition for reproduction, the effect of which is similar to that of the MSR [20,92]. Ancona *et al*. [20] examined 201 bird species, including 12 raptors, seven of which were accipitrids; their results showed that a female bias in ASRs directs the evolution of male-biased SSD and polygamy by males. This would lead males to delay maturation compared to females. The Harpy Eagle does not fit the main model provided by the authors, as it exhibits female-biased ASR and RSD, and the sexual maturity of females occurs later. As the larger sex requires more time to grow, it matures later than the smaller sex [15,93]. While Harpy Eagle females take six years to mature, males take four [67]. As a result, the population MSR is likely to be greater than the SSR and ASR. Additionally, Harpy Eagle females exhibit a shorter reproductive lifespan than males; while females reproduce up to the age of 29 years, males can reproduce up to the age of 35 years [67]. Adding this to the fact that the females invest more time in caring for their offspring [60], the population operational sex ratio (OSR), which is the proportion of sexually active males and females in the breeding pool [94], is probably larger than the ASR. These factors may also offset the higher proportion of females at birth. If males have 31 years to reproduce and females have 23 years, the effective SR of breeding time would be 1.35 : 1. Assuming an adult male SR of 31%, the effective adult male SR would be 42%.

### 4.3. Potential consequences for Harpy Eagle sex roles

Except for the long and more present female parental care in Harpy Eagles, other features of their reproductive behaviour are not as clear. Harpy Eagle is assumed to be socially monogamous based on observations of a pair in the same nest for several reproductive cycles [48,60]. However, we believe that a male Harpy Eagle can potentially assist a female in another nest in the phenology of a distinct reproductive cycle. Although social monogamy is a standard behaviour in birds, most birds exhibit some level of extra-pair mating [95] because of the densities of males and females in the population [96–98]. Excess females are associated with a polygynous system [15,17,20,99].

The association between polygyny and male-biased dispersal in birds and mammals has been discussed [96,100–105]. The high levels of population structure found through the maternal inheritance marker of mitochondrial DNA of the Harpy Eagle in Central and South America by Lenner *et al*. [72], and the low levels of structure found through biparental microsatellite markers by Banhos *et al*. [73] in the Brazilian Amazon Rainforest and Atlantic Forest suggest that males disperse more than females [106].

### 4.4. Sex ratio and environmental changes

In better environmental conditions, the more expensive sex can be produced in high proportions [5,107,108]. Conversely, a longer period of parental investment is more susceptible to environmental unpredictability, which may reduce any sex-based bias in offspring, as it would be more difficult for parents to predict the amount of resources they would have to raise an offspring [9]. In general, the Amazon Rainforest and Atlantic Forest differ in forest quality because of differences in their natural ecological characteristics and history of environmental degradation. The Amazon Rainforest has suffered high rates of deforestation in the last decades of the twentieth century and early decades of the twenty-first century [109,110], more than 20% of its area has already been cleared [111]. By contrast, the primary forest cover of the Atlantic Forest was predominantly deforested throughout the twentieth century, almost 90% of its area has already been cleared [112,113]. Consequently, the conservation status of the Harpy Eagle in the Atlantic Forest is more critical than in the Amazon Rainforest [114]. However, we found no differences in the Harpy Eagle sex ratios between these regions. Comparisons within the regions and over time by life stage reinforced the bias towards a greater number of females than males, although the number of samples collected per period by region was limited. At least within the scale studied, environmental changes over time did not cause changes in the Harpy Eagle sex ratios.

Other studies have pointed to paternal or maternal condition and quality as the reason for bias in SRs, where the more costly sex is produced when one parent is older and in better breeding territories [115–117]. Morandini *et al*. [117], for example, found that age of the breeding parents, but not by territory quality, explains hatching SR in Booted Eagles (*Aquila pennata*), in southwestern Spain, with older breeders more females (the expensive sex). While we have no direct data on the ages of the Harpy Eagle breeding parents, offspring produced in successive nesting periods are produced by successively older parents, and there is no discernible trend in producing more female offspring as function of nesting period. The pairs that had multiple nesting periods also are likely to occupy better breeding territories.

## 5. Conclusion

1. The SR of Harpy Eagles, as indicated by SSR and ASR, exhibits a notable positive female bias, which is likely an outcome of the PSR. This inherent bias could potentially be balanced by the earlier sexual maturation observed in males, implying an extended reproductive lifespan and a higher proportion of sexually active males within the population.
2. The skewed SR towards females may have repercussions on the sex roles within the species, warranting further exploration. Subsequent studies should delve into various facets of the Harpy Eagle's life history and the evolutionary dynamics of sex roles within the population. A comprehensive understanding of these aspects can be achieved through the study of wild populations, as well as through the examination of zoo specimens and museum collections.
3. These findings hold significance for the broader field of ecological evolutionary models in avian species, especially among Accipitridae and birds exhibiting RSD.
4. Incorporating knowledge about the Harpy Eagle's life history into management and conservation strategies is recommended. For instance, the SR data can serve as a valuable input for more precisely estimating effective population size, and it should be integrated into population viability analyses [25] for this species threatened with extinction.

## Data Availability

All relevant data are available within the paper and at https://doi.org/10.5061/dryad.59zw3r2dj [118]. Electronic supplementary material is available online [74].
